# Predicting Antibiotic Resistance and Assessing the Risk Burden from Antibiotics: A Holistic Modeling Framework in a Tropical Reservoir

**DOI:** 10.1021/acs.est.3c10467

**Published:** 2024-04-01

**Authors:** Xuneng Tong, Shin Giek Goh, Sanjeeb Mohapatra, Ngoc Han Tran, Luhua You, Jingjie Zhang, Yiliang He, Karina Yew-Hoong Gin

**Affiliations:** † Department of Civil & Environmental Engineering, National University of Singapore, 1 Engineering Drive 2, Singapore 117576, Singapore; ‡ NUS Environmental Research Institute, National University of Singapore, 1 Create way, Create Tower, #15-02, Singapore 138602, Singapore; § Northeast Institute of Geography and Agroecology, Chinese Academy of Sciences, Changchun 130102, China; ∥ Shenzhen Municipal Engineering Lab of Environmental IoT Technologies, Southern University of Science and Technology, Shenzhen518055,China; ⊥ School of Environmental Science and Engineering, Shanghai Jiao Tong University, Shanghai 200240, China

**Keywords:** antibiotics, antimicrobial resistance, bacteria, integrated modeling, risk assessment

## Abstract

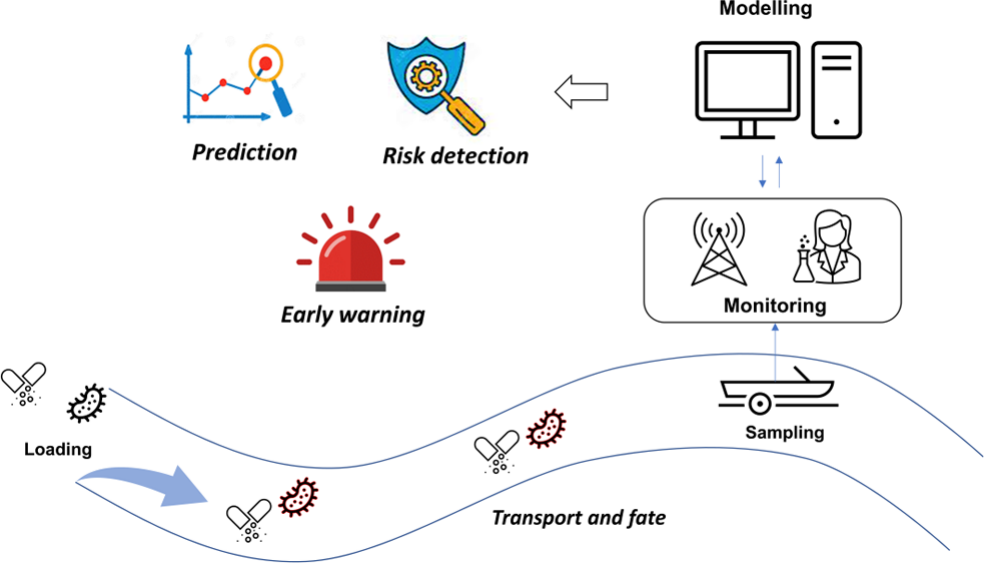

Predicting the hotspots of antimicrobial resistance (AMR) in aquatics is crucial for managing associated risks. We developed an integrated modeling framework toward predicting the spatiotemporal abundance of antibiotics, indicator bacteria, and their corresponding antibiotic-resistant bacteria (ARB), as well as assessing the potential AMR risks to the aquatic ecosystem in a tropical reservoir. Our focus was on two antibiotics, sulfamethoxazole (SMX) and trimethoprim (TMP), and on *Escherichia coli* (*E. coli*) and its variant resistant to sulfamethoxazole-trimethoprim (EC_SXT). We validated the predictive model using withheld data, with all Nash-Sutcliffe efficiency (NSE) values above 0.79, absolute relative difference (ARD) less than 25%, and coefficient of determination (*R*^2^) greater than 0.800 for the modeled targets. Predictions indicated concentrations of 1–15 ng/L for SMX, 0.5–5 ng/L for TMP, and 0 to 5 (log_10_ MPN/100 mL) for *E. coli* and −1.1 to 3.5 (log_10_ CFU/100 mL) for EC_SXT. Risk assessment suggested that the predicted TMP could pose a higher risk of AMR development than SMX, but SMX could possess a higher ecological risk. The study lays down a hybrid modeling framework for integrating a statistic model with a process-based model to predict AMR in a holistic manner, thus facilitating the development of a better risk management framework.

## Abstract 2

Integrated modeling predicts antibiotic levels and hotspots of antimicrobial resistance in aquatic environments, aiding risk assessment and management.

## 1 Introduction

Antibiotics are one of the most commonly used pharmaceuticals in preventing and treating microbial infections^1^ as well as promoting the growth of animals in livestock and aquaculture farms.^2^ Due to their incomplete metabolism in animal and human bodies, antibiotic residues tend to be excreted through feces and urine and subsequently transported to wastewater and receiving water bodies.^3^ The major concern regarding antibiotics in the aquatic environment is increasing resistance in microorganisms to antibiotics, namely antimicrobial resistance (AMR), which has been listed as one of the top 10 global public health threats facing humanity by the World Health Organization (WHO).^4^ In addition, antibiotics have been verified to pose adverse effects on aquatic ecosystems by many *in vitro* or *in vivo* assays.^5−7^

Current wastewater treatment processes are often unable to completely eliminate antibiotics and the associated antibiotic-resistant bacteria (ARB) from effluents.^8^ Consequently, these substances, which contribute AMR, are likely to end up in natural water bodies.^9^ In addition, the presence of antibiotics in the aquatic environment plays an important role in both the transmission and evolution of antibiotic resistance.^10^ It has been acknowledged that more and more bacteria (e.g., *Escherichia coli*, *Pseudomonas putida*, and *Salmonella typhimurium*) have been acquiring resistance to multiple antibiotics in global aquatic environments though a link to human health impacts has not yet been established.^11−13^ In particular, the rapid and widespread increase in antibiotic applications in human and veterinary medical treatments has led to a surging trend of dissemination of antibiotic-resistant bacteria from humans and animals to aquatic environments.^14^ Thus, there is an urgent need to establish and quantify the risk of antibiotics and related AMR issues in aquatic environments.^15^

The first step to mitigate these antibiotics and related AMR issues is to understand their source, transport, fate, and final destination in aquatic environments.^16^ Widespread reports of AMR occurrence in many diverse aquatic environments have already provided early warnings to scientists and policymakers.^17^ However, the analysis of AMR highly depends on skilled expertise and state-of-the-art equipment, which makes the collection of AMR data limited and expensive. In addition, *in situ* sampling is costly, time-consuming, and difficult to sustain for high-frequency and spatial-resolution sampling schemes.^18^ Hence, it is vital to develop novel methods to investigate the transport and fate of AMR in the aquatic environment to overcome the burdens of expensive sampling campaigns and laboratory analysis.^19^ To overcome this limitation, the numerical model can act as a powerful technique to investigate the transport and fate of AMR in aquatic environments^19^ and provide a holistic distribution of AMR over time and space.^16^ The process-based hydrodynamic water quality (HWQ) model that considers the coupled physical–chemical–biological processes (e.g., advection-diffusion, adsorption/desorption, growth/mortality, and grazing processes) of modeled substances is a reliable method to predict the transport and fate of chemical and microbiological pollutants in aquatic environments^20^ and has been widely applied in freshwater and marine ecosystems.^21^ Modeling the fate and transport of AMR (e.g., ARB) in catchments is still at an early stage,^22^ and so far, only a few studies have been conducted to develop such a model.^23,24^ Hellweger et al. introduced a mechanistic model to study tetracycline resistance in aquatic environments, incorporating variables such as antibiotics, bacteria, and organic matter and successfully applied it to the Poudre river.^23^ In their subsequent study, they introduced another model that includes heavy metals like Cu and Zn as influencing factors.^24^ However, these studies were limited by the lack of understanding of how normal bacteria evolve to ARB, thus bringing challenges to developing the fate and transport model of AMR. Nevertheless, evidence in field sampling has verified strong correlations between antibiotics, bacteria, and their related AMR.^17^ Hence, statistical methods, such as multilinear regression (MLR), would be good options for describing the kinetics between antibiotics, bacteria, and AMR.^25^ Eventually, the successful incorporation of AMR kinetics described by the MLR model into the HWQ model would provide new insights into predicting the transport and fate of AMR.

Sulfamethoxazole (SMX) and trimethoprim (TMP) are two common antibiotics, which are mainly consumed at a fixed ratio of 5:1 (SMX:TMP) in a single drug, namely, SXT, for synergistic effects between the two compounds.^26^ The global consumption of SXT was the fourth highest after penicillin, macrolide, and fluoroquinolone as published by WHO.^27^ The current increase in bacteria resistant to SXT necessitates greater concern from the public.^28^*E. coli* is an established fecal indicator bacterium of sewage and animal waste contamination.^29^ In recent decades, the widespread occurrence of *E. coli* resistant to multiple antibiotics in the aquatic environment has received increasing attention because of its applicability as an ARB indicator.^30^ Hence, the successful prediction of *E. coli*-relevant ARB would provide important implications for better management of AMR in aquatic environments. Apart from the emergence of antibiotic resistance, antibiotics also have been regarded as emerging chemical contaminants because of their longer environmental persistence^31^ and their potential toxicity on aquatic ecosystems.^32^ Hence, a coupled risk assessment considering both the development of AMR and their ecological toxicity in the aquatic environment would be a more realistic way to evaluate the environmental risks due to the occurrence of antibiotics.

The objective of this study aims to lay down an integrated modeling framework to predict the spatiotemporal distributions of antibiotics, indicator bacteria, and corresponding ARB that can be used to identify the hotspots of AMR and assess the potential environmental risks from antibiotics in the aquatic environment. In this study, two representative antibiotics (SMX and TMP), indicator bacteria (*E. coli*), and their relevant ARB (EC_SXT) were modeled. The new insights revealed by the integrated modeling framework can act as a useful early warning AMR toolbox to identify the spatiotemporal hotspots of AMR with regard to their levels and risks.

## 2 Materials and Methods

### 2.1 Sampling and Environmental Data

The study area is a highly urbanized reservoir fed with five tributaries (Figure S1, Supporting Information). The main functions of the reservoir are to harvest rainfall water for drinking water production, flood control, as well as for recreational use (i.e., kayaking and boating, etc.).^33^ However, given that the studied reservoir is located in the downtown commercial district, it is also susceptible to anthropogenic pollutants. A detailed description of the study area has been provided in our previous studies.^34,35^ The sampling campaign was conducted from Dec 2015 to Sep 2016 at five tributaries and one location within the reservoir (hereinafter referred to as the “main water body”, indicated as S1), which is shown in Figure S1. To provide a clearer understanding of our model’s comprehensive data usage, it is crucial to differentiate between the data collected from the tributaries and that from within the reservoir. Specifically, monthly data from the tributaries were vital for defining the loading parameters for our model’s open boundaries (Figure S1), which includes 30 open boundaries with 10 data points each, totaling 300 data points used in model development. This distinction was essential to ensure accurate simulations of water system dynamics. On the other hand, the data obtained directly from the reservoir were instrumental in the model validation process. Compared with the loading data set, our validation data set for the HWQ-AMR model is limited, with 6 data points of SMX, TMP, and *E. coli* and 4 data points of EC_SXT due to experimental constraints. This dual approach in utilizing distinct data sets significantly enhanced the model’s accuracy and reliability. A detailed description of the data set used for the open boundaries is provided in the Supporting Information. Furthermore, it is important to highlight the validated use of data from our previous studies, notably those by Wang et al.^36^ and Tong et al.^37^ These studies rigorously verified the efficacy of the data sets in calibrating and validating various aspects of our model, including hydrodynamic characteristics, general water quality parameters, eutrophication processes, and the behavior of emerging organic contaminants. The successful application of these data sets is found in prior research.

In our study, water samples for chemical and bacterial analyses were specifically collected from the surface layer at a depth of 0 to 0.2 m despite our HWQ-AMR model’s capability to include deeper layers and sediments. This approach was informed by the characteristics of our study area, a typical shallow reservoir where water is well-mixed throughout the water column. Such mixing suggests that the surface water effectively represents the overall conditions of the reservoir. For each type of analysis (antibiotics and bacteria), we used two separate 1.0 L amber plastic bottles: one for antibiotics (SMX and TMP) and another for bacterial (*E. coli* and EC_SXT) analysis. During the analysis of water samples, 500 mL water samples were processed for each batch, and 1 L of water samples was required for duplicate evaluations. This sampling strategy, focusing on the surface layer, was deemed sufficient for accurately assessing the presence and levels of our target substances in the well-mixed reservoir environment. Antibiotics (SMX and TMP, in units of nanograms per liter) in the dissolved phase were analyzed by a combination of solid phase extraction and ultrahigh-performance liquid chromatography-tandem mass spectrometry (SPE-UPLC/MS/MS). The abundance of *E. coli* in units of most probable number (MPN)/100 mL was measured with *Colilert* (*IDEXX Laboratories, Inc., Westbrook, Maine*), respectively, according to the manufacturer’s instructions. Culture-based methods were used to screen for *E. coli* against cotrimoxazole (EC_SXT, CFU/100 mL), an antibiotic consisting of two different antibiotics (SMX and TMP). During the analysis of water samples, at least one procedural blank duplicate and one matrix spike duplicate were processed for each batch. The detailed analytical methods for the measurements of antibiotics and bacteria can be found in the Supporting Information and our previous studies.^33,38^

### 2.2 Coupled Hydrodynamic Water Quality Antimicrobial Resistance Model

A coupled hydrodynamic water quality antimicrobial resistance model was developed based on the Delft 3D software suite (http://oss.deltares.nl/web/delft3d) to simulate the transport and fate of antibiotics, *E. coli*, and ARB within the main water body. First, the hydrodynamic conditions were computed both in horizontal and vertical directions by a 3D hydrodynamic model (Delft 3D-Flow),^39^ which was later calibrated and validated based on a historical data set including parameters such as flow velocities, water levels, salinity, and temperature data from previous studies.^36^ As stated earlier, the results from the hydrodynamic model were delivered to the water quality-AMR model, which comprises the state variables of general water quality parameters, phytoplankton, antibiotics, *E. coli*, and ARB. The simulated general biogeochemical water quality parameters (e.g., total nitrogen (TN), total phosphorus (TP), chlorophyll-a (Chl-a), dissolved oxygen (DO), total organic carbon (TOC), and total suspended solids (TSS)) and biomass of phytoplankton species in the water quality module have been rigorously validated in our previous modeling work.^37^ The water quality model performance was evaluated using statistical metrics such as RMSE, ARD (%), and NSE (Figure S2). The results indicated a good fit with the historical data set, which has been reported in our previous studies,^20,37^ with NSE values ranging from 0.31 to 0.91 and ARD (%) values below 25, demonstrating that the HWQ model can realistically capture the fluctuations of hydrodynamics water quality within this water body.

**Figure 1:**
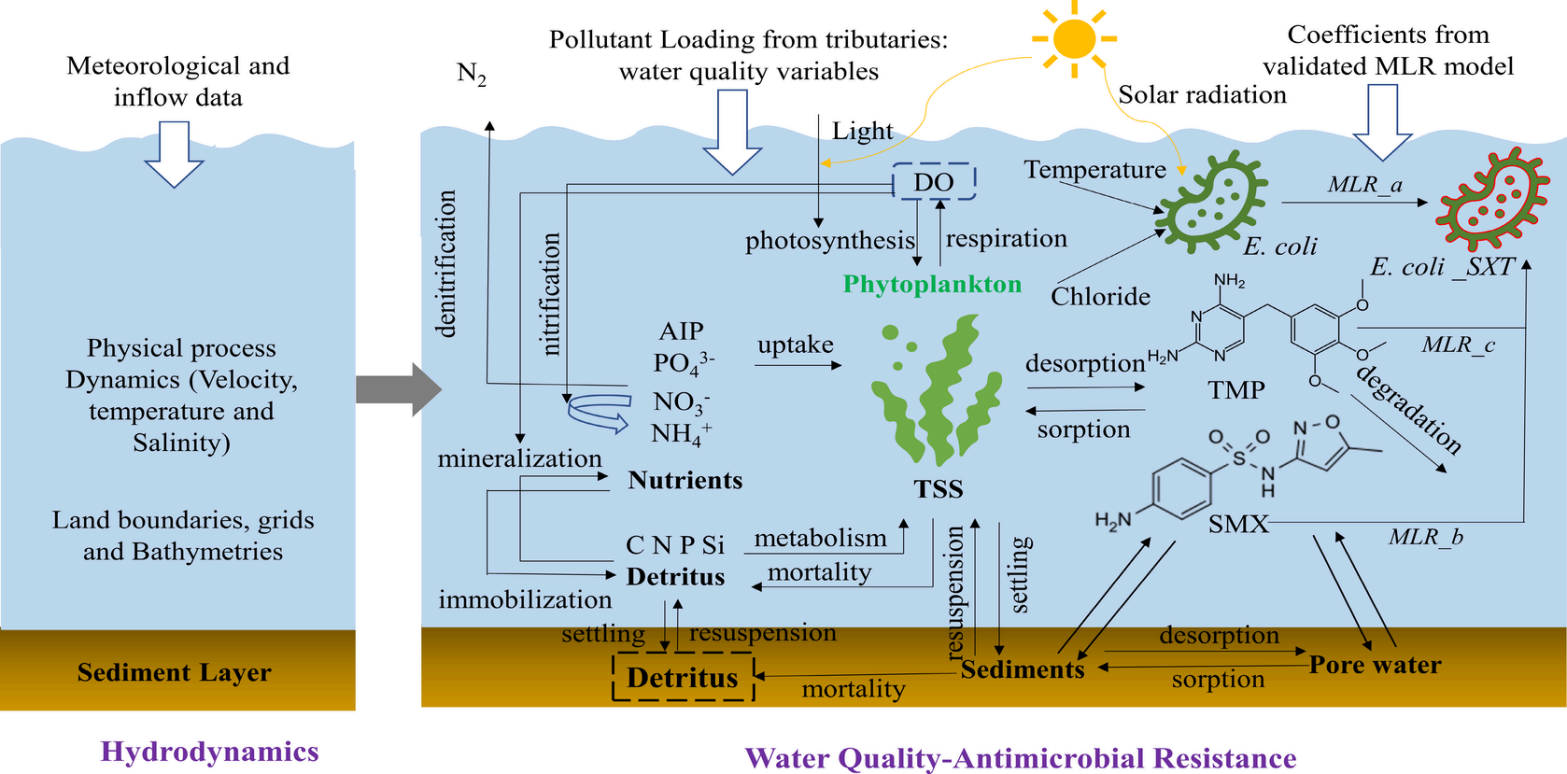
Structure and key processes of the HWQ-AMR model.

In this study, we further expanded the HWQ model to incorporate the processes of bacteria, antibiotics, and ARB into the new HWQ-AMR model. Sampling data from the main water body was used to calibrate and validate the HWQ-AMR model. The simulation period was from the first of October 2015 to the first of October 2016, covering the entire sampling period. The model structure and key processes of the HWQ-AMR are shown in Figure 1. Here, we present the essential descriptions of the AMR modules (antibiotics, bacteria, and ARB module) in the HWQ-AMR model, while the hydrodynamic module, advection-diffusion-reaction processes, eutrophication module, full set of equations, parameters values, and references are provided in Supporting Information.

#### 2.2.1 Antibiotics Module

The kinetic processes of antibiotics in the aquatic environment are influenced by organic matter (i.e., dead particulate organic matter, phytoplankton, and dissolved organic matter) which act as the main media for the sorption and partitioning of antibiotics.^40^ The temporal and spatial concentrations of detritus-dissolved organic matter and phytoplankton were calculated by the water quality model. Partitioning is the process by which a substance is distributed among various dissolved and absorbed species.^41^ The partitioning of antibiotics is described as an equilibrium process by means of a linear partition coefficient based on the amounts of organic carbon, and the sorption flux is calculated according to equilibrium partitioning.^42^ The model simulates the total concentration (i.e., total particulate and total dissolved concentrations) of each antibiotic, where the volume unit refers to bulk water. The partitioning process delivers the dissolved and adsorbed species as fractions of the total concentration and the sorption flux. In addition, one sediment layer consisting of pore water and sediments was integrated into the model. In the sediment layer, all substance quantities are converted into bulk concentrations by dividing the volume of the layer. The settling and resuspension processes of antibiotics were coupled to the settling and resuspension processes of particulate organic detritus and algal biomass. The settling rates of all individual carrier substances are generated by the process as the sum of zero- and first-order kinetics. The key processes and governing equations are summarized in Table S2. Representative calibrated values of the key kinetic processes are summarized in Table S3.

#### 2.2.2 Bacteria Module

The model results from the hydrodynamic and eutrophication modules are coupled with those of the bacteria module. Bacteria mortality is governed by the processes of UV radiation, chloride, temperature, and their inherent mortality. The formulations for the bacteria modules are mainly empirical, and the mortality rate was based on a first-order reaction extracted from Mancini et al.’s study.^43^ Our model elucidates the nuanced interplay between bacterial dynamics and environmental factors. We incorporate temperature, chloride, and solar radiation, with the latter’s influence modulated via Secchi disk depth, an algae-influenced parameter. This depth integrates key water quality determinants—(in)organic suspended matter, chlorophyll, and dissolved organics (fulvic and humic acids), subtly embedding nutrient impacts on bacterial behavior. Supplementary Figure S3 articulates these relationships, demonstrating how water quality, particularly phosphorus and chlorophyll-a levels, indirectly but significantly influences bacterial persistence through Secchi depth modulation. The key processes are summarized in Table S2. Representative calibrated values of the key kinetic processes are summarized in Table S3.

#### 2.2.3 ARB Module

The relationship between antibiotics (SMX and TMP), bacteria, and ARB is described by multiple linear regression (MLR). The MLR model was developed using the scikit-learn package in Python (version 3.8).^44^ In evaluating the fate and transport of ARB, the primary focus is on the resistance development pressure induced by specific antibiotics.^45−47^ Additionally, we have incorporated crucial environmental factors, such as salinity and temperature, which significantly influence the fate of bacteria.^48,49^ By incorporating these environmental parameters, our model simulates the ARB dynamics influenced by these conditions, ensuring a more accurate and holistic understanding of their fate in aquatic environments. The predictors chosen for the MLR model are two antibiotics, SMX and TMP, along with *E. coli*, with the prediction target being its corresponding ARB (EC_SXT). The rationale behind selecting these specific predictors is grounded in their established roles within the AMR mechanisms. Specifically, the antibiotic residues of SMX and TMP play crucial roles in influencing antibiotic susceptibility and the evolution of resistance. Additionally, the presence and concentration of *E. coli* are vital markers for bacterial interactions with other parameters. We utilized a data set comprising 14 sets, totaling 64 data points. While seemingly modest, this data set size was sufficient to capture the necessary variability and relationships between SMX, TMP, *E. coli*, and EC_SXT. Bacteria colonies were normalized by log_10_ transformation, which is commonly used in the description of bacteria abundance.^50^ The concentrations of antibiotics (SMX and TMP) and the normalized value of the *E. coli* abundance from the samplings are used as inputs to the MLR model. The normalized values of EC_SXT abundance are obtained as the model outputs. The basic form of the MLR model is
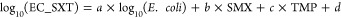
1where EC_SXT is expressed in CFU/100 mL, *E. coli* is expressed in MPN/100 mL, and SMX and TMP are expressed in ng/L; *a*, *b*, and *c* is the coefficient for *E. coli*, SMX, and TMP, respectively, and *d* is a *y*-intercept.

The established MLR model was tested using field data and evaluated using standard statistical methods to assess its performance rigorously. Detailed information about these evaluation methods is provided in Section 2.4.

In our integrated modeling approach, a crucial kinetic linkage exists among the antibiotics, bacteria, and ARB modules, ensuring a dynamic and interconnected representation of the factors influencing ARB in aquatic environments. Initially, the MLR model was meticulously trained and validated using field data collected from tributaries and the main water body, ensuring the model’s robustness and applicability to real-world scenarios. Once the model is validated, the coefficients derived from the MLR model are incorporated into the governing equations of the ARB module. This step is critical, as it bridges the theoretical understanding with empirical data, allowing the model to reflect real-world dynamics accurately. The ARB module, thus armed with these coefficients, becomes adept at simulating the relationships and interactions between antibiotics, bacteria, and ARB. The outputs from the antibiotics and bacteria modules—specifically, the computed concentrations of antibiotics and bacteria—serve as vital inputs into the ARB module. The antibiotic module provides data on the concentration and behavior of antibiotics in the aquatic environment, factoring in processes like sorption, partitioning, settling, resuspension, and so on. Concurrently, the bacteria module contributes insights into bacterial populations influenced by environmental conditions such as temperature, solar radiation, and so forth. The ARB module synthesizes these inputs, applying the MLR coefficients to compute the concentration of ARB. This computation is not just a mere aggregation of data but a sophisticated analysis that considers the kinetics of antibiotic behavior, bacterial mortality, and the resultant emergence of ARB. Such a comprehensive approach ensures that our model accurately reflects the complex interplay of biological and chemical processes in aquatic systems, providing a reliable tool for predicting the concentration and behavior of ARB.

### 2.3 Environmental Risk Assessment

The environmental risk assessment of antibiotics was considered in light of two aspects: (1) the development of AMR and (2) the ecological toxicity in the aquatic environment. First, the potential risk of antibiotics to AMR development was evaluated via the ratio between the predicted antibiotic environmental concentration (computed environmental concentration (CEC), in the unit of ng/L) from the HWQ-AMR model and the predicted no-effect concentrations for resistance selection (PNEC_AMR_, in unit of ng/L) taken from the literature and as depicted in eq 2. The potential risk of antibiotics to the aquatic ecosystem was evaluated via the ratio between the computed environmental concentration (CEC, in units of ng/L) from the HWQ model and the no-effect concentration for the ecosystem (PNEC_Eco_, in units of ng/L) taken from the literature and as depicted in eq 3. The PNEC_AMR_ values were obtained from the literature, that is, estimated from the upper boundaries of the selective concentrations in the targeted antibiotics based on the assumption that selective concentrations *a priori* need to be lower than those completely inhibiting growth.^51^ Similarly, the PNEC_Eco_ values collected from the literature were calculated based on the lowest value of ecotoxicological data from the existing data set, that is, the selective concentrations in the targeted antibiotics that could inhibit the growth of the ecotoxicological bioassay indicator (e.g., microalgae).^52^ The detailed calculation methods for the PNEC values are provided in the Supporting Information.
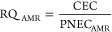
2
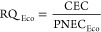
3where RQ_Eco_ and RQ_AMR_ are the risk quotients to the ecosystem and AMR development, respectively. The RQ ranking criteria were applied to interpret the classifications of risks as “unlikely to pose risk”: RQ ≤ 0.01; “low risk”: 0.01 < RQ ≤ 0.1; “medium risk”: 0.1 < RQ ≤ 1; “high risk”: RQ > 1.^53^

### 2.4 Model Evaluation

The performance of the models was evaluated by RMSE, ARD (%), NSE, and *R*^2^, as defined in the Supporting Information. The levels of the model performance were categorized as NSE > 0.65 excellent simulation; 0.5–0.65 very good; 0.2–0.5 good; < 0.2 poor.^36,54^ As the ARD% is less than 25%, the model performance is considered satisfactory.^40,55^

## 3 Results and Discussion

### 3.1 Relationship of Antibiotic Resistance Bacteria Revealed by MLR

The development of the MLR model is deeply rooted in understanding the complex mechanisms of AMR. These mechanisms include factors like antibiotic residue,^56^ bacterial interactions,^57^ evolution,^58^ and decay,^59^ which play a crucial role in the persistence, degradation, and propagation of antibiotic resistance in aquatic environments.^60^ Our model focuses specifically on three predictors: two antibiotics (SMX, TMP) and *E. coli*, in relation to their corresponding ARB (EC_SXT). The choice of these predictors is substantiated by their significant roles in the AMR mechanisms. For instance, SMX and TMP antibiotic residues are critical in influencing antibiotic susceptibility and resistance evolution.^61^ The presence and concentration of *E. coli* serve as a marker for bacterial interactions and growth rates, factors that are pivotal in understanding ARB dynamics in aquatic environments.^62^ The developed MLR model was fitted with a slope for each independent variable and expressed in eq 4:
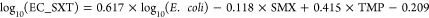
4

**Figure 2:**
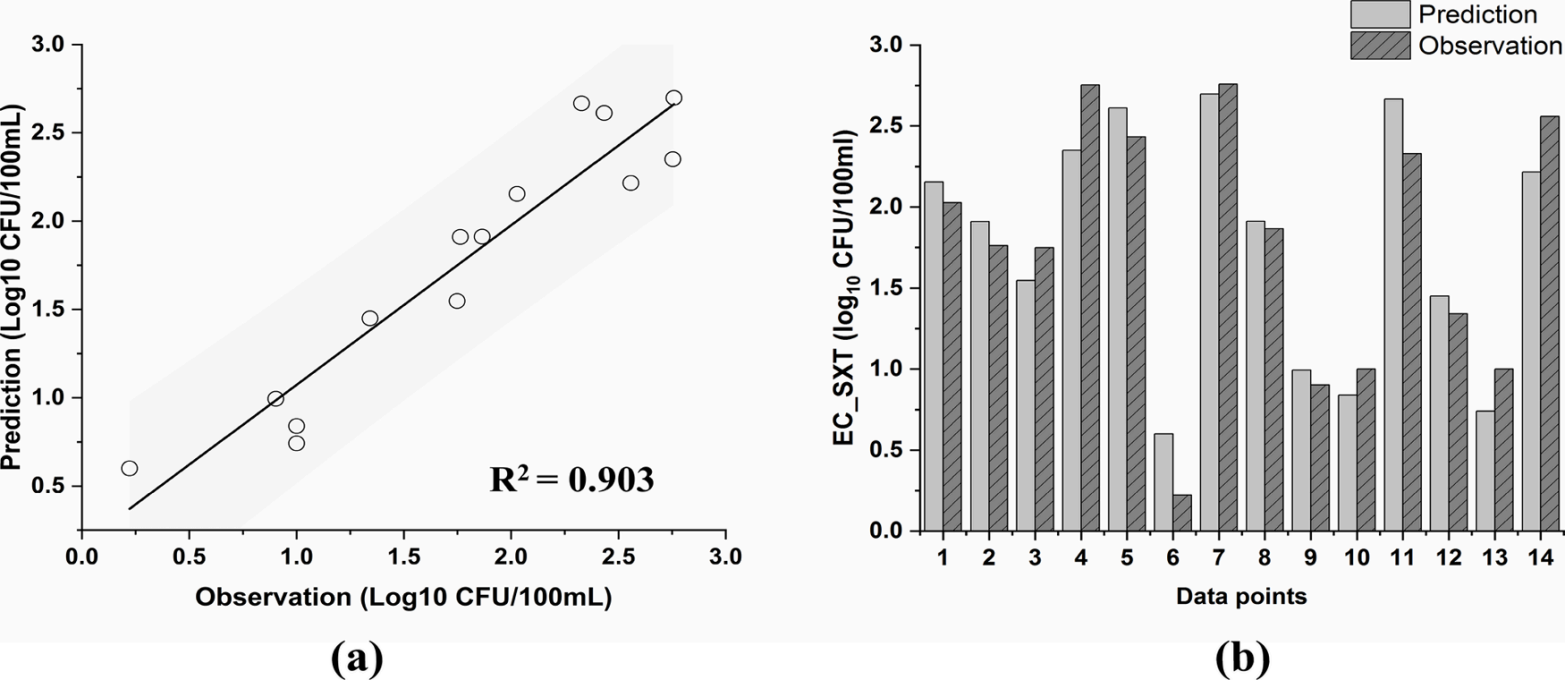
Comparison of the observations versus MLR predictions. (a) Linear regression evaluation: the solid line represents a line of perfect agreement between the observations and the predictions, the shaded area represents 95% of the prediction band, and (b) bar chart along the data points.

The MLR model, as indicated by a high *R*^2^ value of 0.903 (Figure 2a), effectively captures the relationship between these predictors and the abundance of EC_SXT. The coefficients assigned to each predictor in the model (eq 4) reflect their varying impacts. The positive coefficient for TMP (*c* = 0.415) and *E. coli* (*a* = 0.617) suggests a direct influence on increasing EC_SXT abundance, aligning with the established understanding of antibiotic residue impacts and bacterial growth in AMR proliferation. Conversely, the negative coefficient for SMX (*b* = −0.118) indicates a limited effect, which aligns with the higher SMX threshold for AMR resistance compared to TMP, a fact supported by Bengtsson–Palme and Larsson’s study.^51^ Hence, for SXT, as a combination of SMX and TMP, the threshold of SXT to AMR resistance depends largely on TMP over SMX, which previous studies have verified.^51,52,63,64^

Moreover, the statistical analysis through MLR further validates these relationships (Table S5). While the intercept is not statistically significant (*t*-value of −0.696, *p*-value of 0.502), the predictor variables SMX and TMP exhibit *t*-values of −2.021 and 1.861 with *p*-values of 0.071 and 0.092, respectively. Although these *p*-values are slightly above the standard cutoff for statistical significance (0.05), they are close enough to suggest a potential influence on the dependent variable, EC_SXT. The negative *t*-value for SMX hints at an inverse relationship, whereas the positive *t*-value for TMP suggests a direct relationship with EC_SXT. The confidence intervals for both variables are narrow and do not vastly exceed the zero bound, implying that their effects could be meaningful in the model context. The log_10_ (*E. coli*) variable, with a *t*-value of 8.063 and a *p*-value close to zero, shows a strong positive influence on ARB, as evidenced by a 95% confidence interval entirely above zero. This significant result for *E. coli* underscores the potential that even variables with marginal *p*-values such as SMX and TMP may have substantive impacts on antibiotic-resistant bacteria when interpreted in a broader context. These results are statistically significant and biologically relevant, considering the aforementioned AMR mechanisms. The comparison between predicted and observed EC_SXT concentrations (Figure 2b) showed that the difference is relatively small. The RMSE (in the unit of log_10_ CFU/100 mL) value was 0.234, and the model performance rating was “excellent” where the NSE value was 0.95 and the ARD (%) was 22. Hence, model performance evaluated via the aforementioned metrics showed that the developed MLR can predict the abundance of EC_SXT. Overall, the model’s excellent performance, indicated by a low RMSE value and a high NSE, along with a comparison of predicted and observed EC_SXT concentrations, solidifies its ability to predict AMR patterns. This integration of statistical robustness and biological relevance underscores the model’s capacity to effectively capture the dynamics of antibiotic resistance in aquatic environments, based on monitored data of antibiotics and indicator bacteria.

### 3.2 HWQ-AMR Model Evaluation and Seasonal Fluctuations

**Figure 3:**
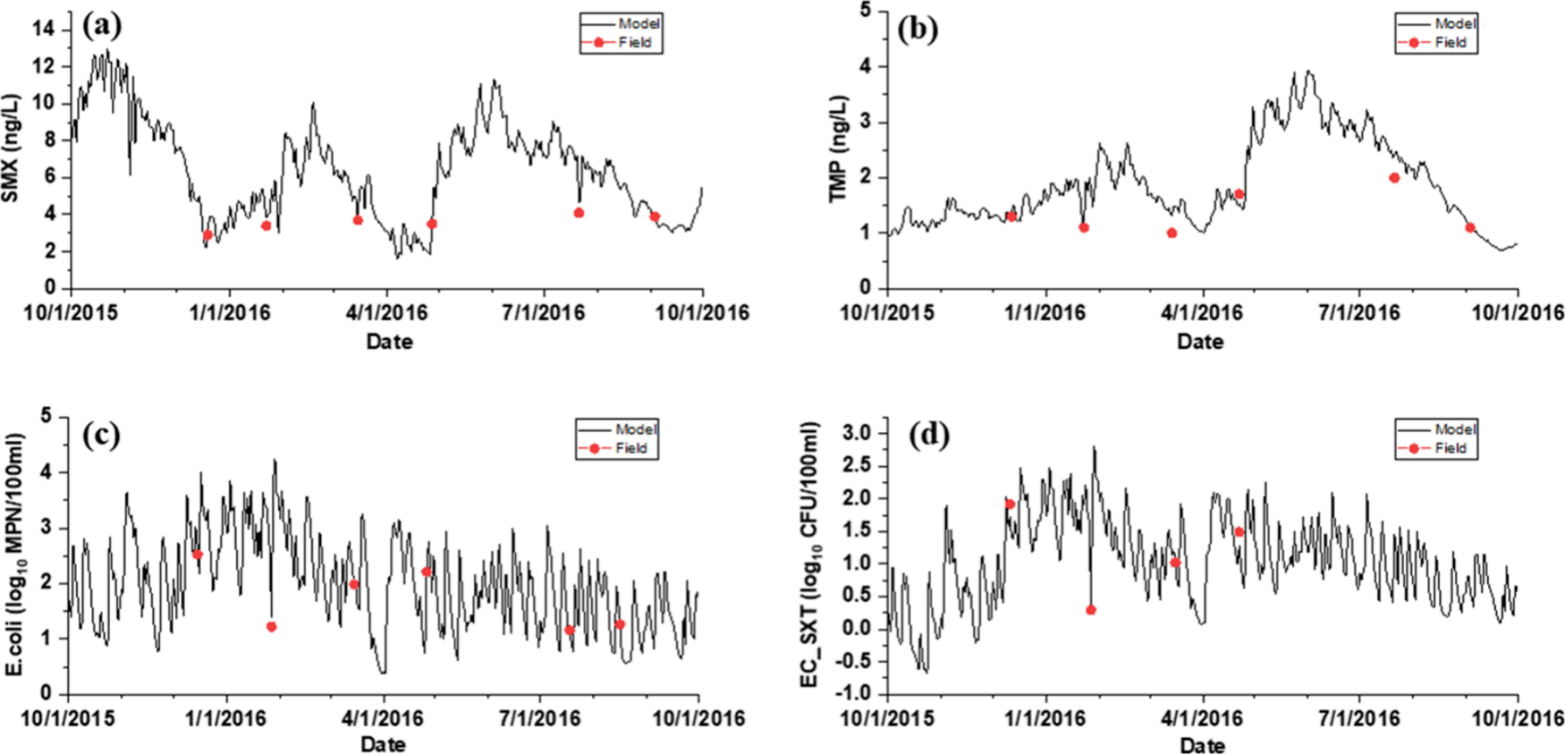
Time series comparison between model results and field data within the water body (S1): (a) SMX; (b) TMP; (c) *E. coli*; and (d) EC_SXT from the HWQ-AMR model.

The HWQ-AMR model was applied to the studied reservoir. Field data of SMX, TMP, *E. coli*, and EC_SXT (from October 2015 to October 2016 within the main water body) were used to compare with the HWQ-AMR model results (Figure 3), and the RMSE, ARD (%), and NSE were used to evaluate the model performance (Table 1). The RMSE (ng/L) values of the modeled SMX and TMP were 0.305 and 0.227, respectively, while the RMSE of modeled *E. coli* (Log_10_ MPN/100 mL) and EC_SXT (Log_10_ CFU/100 mL) was 0.106 and 0.011, respectively. The NSE values of modeled SMX, TMP, *E. coli*, and EC_SXT were 0.79, 0.85, 0.98, and 0.98, respectively. Also, all ARDs (%) of the modeled substances were smaller than 25. The *R*^2^ values of modeled targets ranged from 0.800 to 0.994. The predicted substances over time and space from the coupled HWQ-AMR results suggest a good match with the observations. Hence, the developed HWQ-AMR model was justified to predict the abundance of EC_SXT and capture the trend in the fluctuations.

**Table 1:** Evaluation Metrics for the MLR and HWQ-AMR Models^a^

model	variables	metrics	value
MLR	EC_SXT	RMSE	0.234
		ARD (%)	22
		NSE	0.95
		*R*^2^	0.903
HWQ-AMR	TMP	RMSE	0.247
		ARD (%)	12
		NSE	0.85
		*R*^2^	0.804
	SMX	RMSE	0.305
		ARD (%)	6
		NSE	0.79
		*R*^2^	0.800
	*E. coli*	RMSE	0.106
		ARD (%)	5
		NSE	0.98
		*R*^2^	0.970
	EC_SXT	RMSE	0.011
		ARD (%)	23
		NSE	0.98
		*R*^2^	0.994

a The units of RMSE are log_10_ CFU/100 mL for EC_SXT; log_10_ MPN/100 mL for *E. coli*; and ng/L for TMP and SMX.

**Figure 4:**
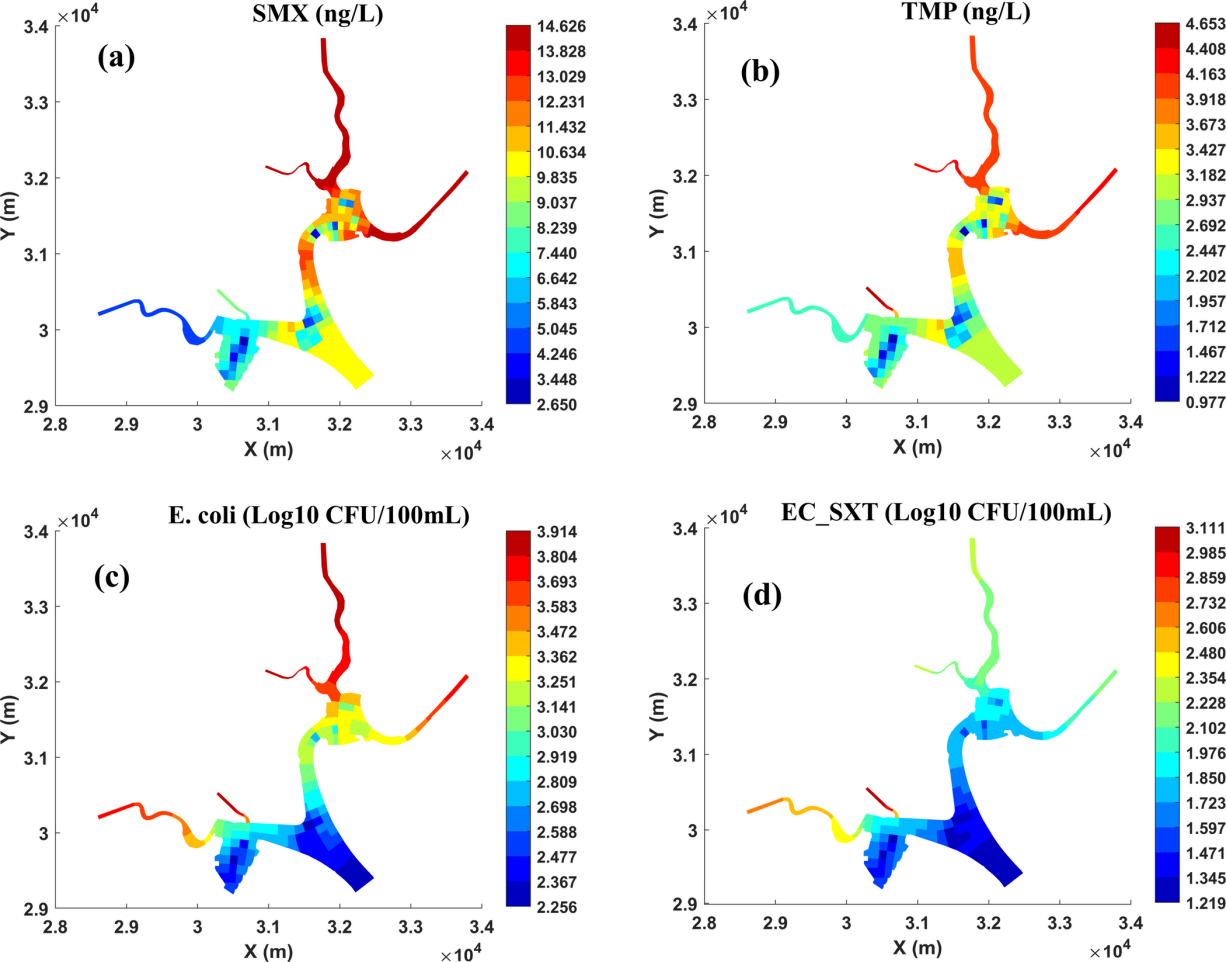
Spatial distributions of modeled substances (for 1st June 2016): (a) SMX; (b) TMP; (c) *E. coli*; and (d) EC_SXT from the HWQ-AMR model.

The results also demonstrated that the predicted substances show spatiotemporally dynamic distributions (Figures 3 and 4). Based on the monthly averaged scale, there were three peaks having SMX levels over 10 ng/L in October 2015, March 2016, and May 2016. Also, the TMP level reached its highest level in May 2016. Notably, March 2016 set a new record for the driest March, with only 6.2 mm of rainfall recorded in Singapore due to the strong El Niño.^65^ However, the strongest 200 mm was observed in May 2016 throughout the study period. In March, the relatively low rainfall of 6.2 mm may not have been sufficient to effectively dilute and disperse the pollutant, potentially leading to higher concentrations. In contrast, the strong rainfall in May (200 mm) could have initially washed pollutants from various sources into water bodies, causing a temporary spike in SXT levels. Generally, the overall fluctuations of SMX (Figure 3a) and TMP (Figure 3b) were similar. The spatial distribution of modeled substances on the first of June 2016 was selected for analysis. This is because the peak value of antibiotic resistance concentration was observed on this date, thus providing the upper boundary of potential risks (AMR development and ecological) caused by antibiotics in the study area. From a spatial distribution point of view, the SMX and TMP levels in the northern part of the study area were higher than those in the rest of the region, especially in the three tributaries in the upper region (Figure 4a,b). Due to the proximity of the upper region to higher-density residential and commercial areas in the downtown area, these three tributaries are more impacted by human activities.^20,37^ In addition, lower dilution factors with smaller water inflow volumes in the tributaries could induce higher concentrations of antibiotics.^33^ Since SMX and TMP are frequently used in association with SXT for medication purposes, the combination ratio generated a unique index for source tracking.^67^ The biodegradability of SMX/TMP evaluated by the MITI test is 0.16 (SMX) and 0.006 (TMP), respectively,^68^ where a chemical is considered as a biodegradable compound if its biodegradability >0.5.^69^ Hence, the SMX/TMP ratio can be considered as a reliable chemical marker for source tracking due to their long-term persistence.^70^ From our model results, the predicted range of the concentrations of SMX and TMP was 1–15 and 0.5–5 ng/L, respectively, which follows the environmental ratio (SMX:TMP) found in the literature,^38,67^ indicating that our model can also act as a powerful source tracking toolbox at the catchment scale.

The predicted log_10_ concentrations of *E. coli* and EC_SXT ranged from 0 to 5 MPN/100 mL and −1.0–3.5 CFU/100 mL (Figure 3c,d), respectively. Also, a similar daily fluctuation between *E. coli* and EC_SXT was observed since the abundance of EC_SXT is mainly determined by the *E. coli* level.^71^ The average percentage of predicted *E. coli* resistant to SXT (EC_SXT) was 17.5%, consistent with the observed historical data reporting an average percentage of 17.4% for EC_SXT in the same catchment.^38^ Compared with global studies, previous field measurements reported that 22% (France),^72^ 13% (Austria),^73^ 17% (US),^74^ and 19% (China)^75^ of *E. coli* were resistant to SXT, in agreement with our predicted results. The daily variation in *E. coli* and EC_SXT abundance is relatively large since the survival of bacteria is determined by multiple parameters (e.g., temperature, salinity, and UV radiation) simultaneously, resulting in complex daily fluctuations.^76^ In addition, the spatial distributions of *E. coli* and EC_SXT (Figure 4c,d) were different from the antibiotics’ pattern (Figure 4a,b). In general, lower levels of *E. coli* and EC_SXT were predicted in all five tributaries, and there was a big difference between the levels in the tributaries and in the main water body. This contrasts sharply with the spatial distribution of antibiotics, where elevated levels were predicted both in the tributaries and in the main water body. This is probably due to the fact that SMX and TMP are persistent organic chemicals that are resistant to degradation in the environment.^31^ As already mentioned, the biodegradability of SMX and TMP is both smaller than 0.5,^69^ indicating they would be persistent in the aquatic environment. As such, their distribution patterns are mainly determined by hydrodynamic conditions overriding the impact of biochemical processes.^16^ In contrast, *E. coli* and EC_SXT, as enteric bacteria, are less likely to survive in natural environments in the long term compared with persistent antibiotics.^77,78^ This is because these bacteria are easily inactivated by sunlight and^79^ temperature^80^ and can adsorb to particles.^81^

### 3.3 Environmental Risk Assessment of Antibiotics

**Figure 5:**
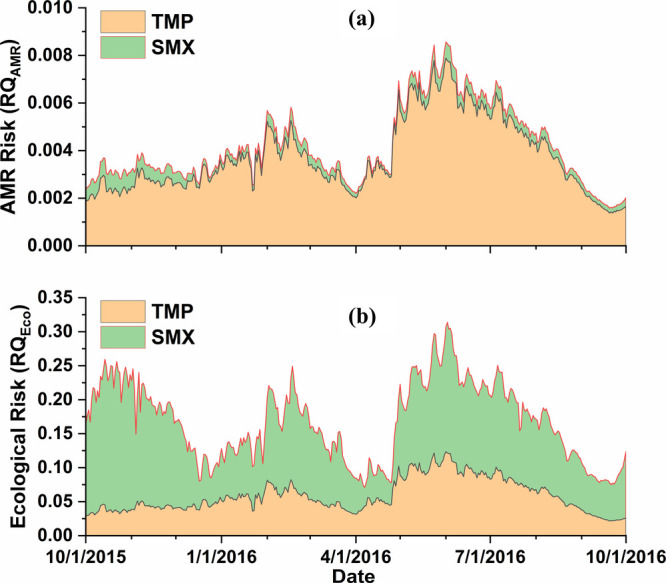
Temporal distribution of predicted environmental risk; the size of stacked area represents risk level; (a) AMR risk; (b) ecological risk.

**Figure 6:**
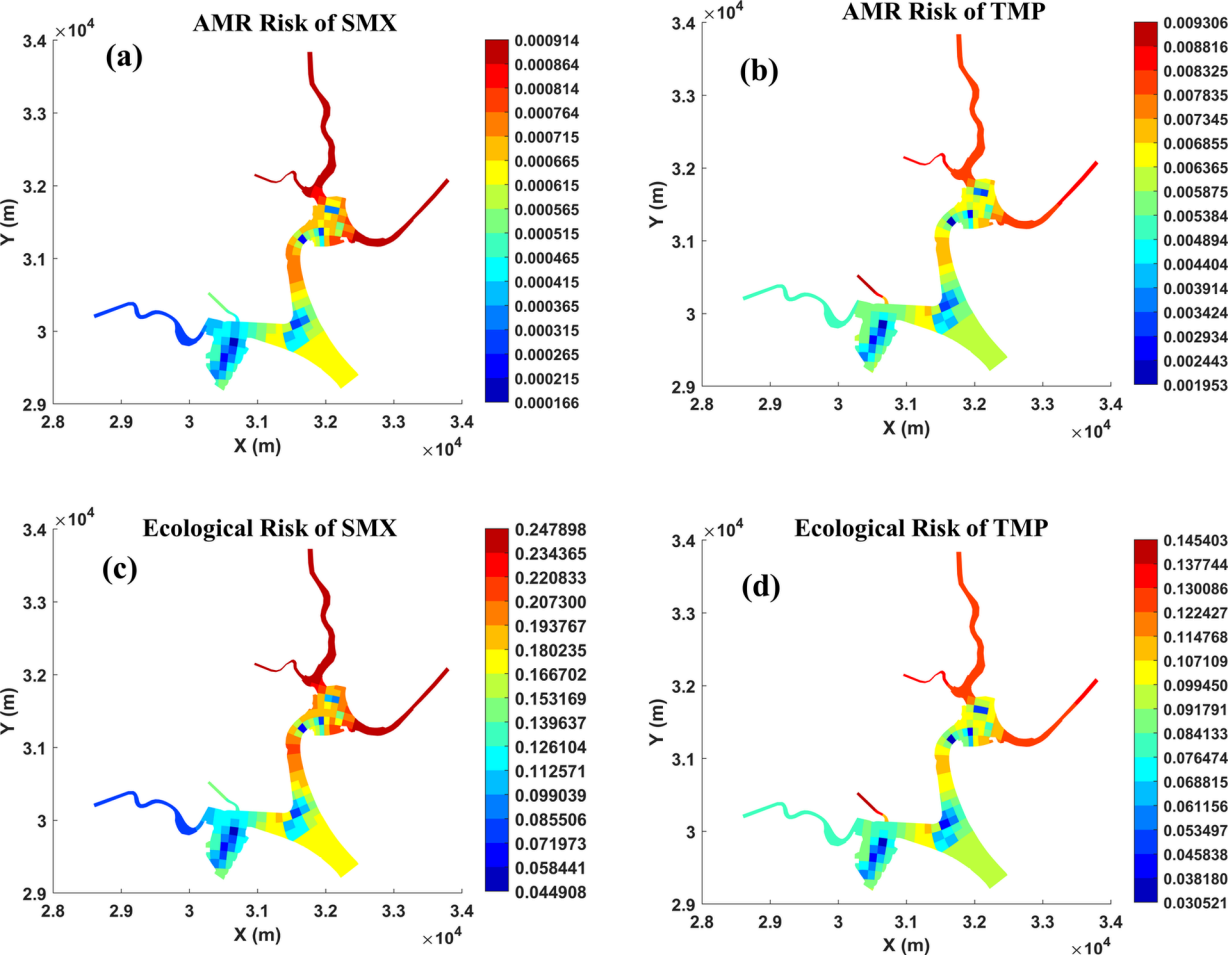
Spatial distribution of environmental risks (for 1 June 2016): (a) AMR risk of SMX; (b) AMR risk of TMP; (c) ecological risk of SMX; and (d) ecological risk of TMP.

The predicted potential environmental risks caused by antibiotics are shown in Figures 5 (temporal distribution) and 6 (spatial distribution). We selected the PNEC_AMR_ for SMX and TMP as 16,000 and 500 ng/L, respectively, based on the estimation from Bengtsson-Palme and Larsson’s study.^51^ The predicted RQ_AMR_ ranged from 0.0001 to 0.0007 (SMX) to 0.0014–0.0079 (TMP). This is consistent with previous studies, which reported that TMP and not SMX determines the AMR risk boundary of SXT.^51^ Although the magnitude of AMR risk predicted by modeling TMP was nearly 10-fold higher than that of SMX (Figure 5a), the current levels of both SMX and TMP do not pose a risk to AMR development. Only the RQ_AMR_ of TMP in the upper catchment region (Figure 6c) was predicted to be close to 0.1, indicating a potential approach to the lower boundary of the AMR risk. The PNEC_Eco_ values for SMX (59 ng/L) and TMP (32 ng/L) were selected based on ecotoxicological data, as calculated by Tran et al.^52^ In contrast to the predicted AMR risk, the predicted ecological risk from SMX is higher than that from TMP throughout the entire simulation period (Figure 5b). The predicted ecological risk (RQ_Eco_) from the selected antibiotics ranged from around 0.04–0.25 (SMX) and 0.03–0.15 (TMP), indicating that the currently selected antibiotics are predicted to pose a low and/or medium risk to the aquatic ecosystem. The higher predicted ecological risk from SMX (compared with TMP) also explained why the MLR coefficients for SMX and TMP were −0.118 (*b* in eq 4) and 0.415 (*c* in eq 4), respectively. This is attributed to the higher ecological (ecotoxicological) burden from ambient SMX levels that could potentially inhibit the growth of *E. coli*.^82^ Hence, the combined influence from an unlikely AMR risk but higher ecological (ecotoxicological) risk from SMX made its coefficient (*b*) negative. This is also supported by current NOEC values presented in FASS (Pharmaceutical Specialties in Sweden, http://fass.se; 2015-08-26) based on ecotoxicological data; that is, there is a lower NOEC boundary of SMX (5.9 μg/L) compared with TMP (56 μg/L).^51^

### 3.4 Limitations and Implications

There are several limitations in the current study. First, a sparse and low-frequency data set was used to develop and validate our models; it would have been preferable to have a higher frequency sampling scheme at finer spatial resolution and with a wider variation in the observed values to deliver a rigorous data set with sufficient data for the proposed modeling framework.^16^ To overcome the technical burdens of environmental analysis, advanced biochemical sensors can be applied in future field sampling programs to collect more data conveniently.^83^ In addition, a large data set could provide diverse modeling choices, such as applying machine learning algorithms to explore the relationship between antibiotic resistance and the surrounding environment.^19^ Beyond the constraints imposed by data set size, establishing sustainable monitoring programs is crucial for providing a valid database essential for water quality model development and achieving expected results. In recent decades, a wealth of field studies on AMR in aquatic environments has emerged.^84,85^ However, few field studies considered the possibility of further building a model when they planned their sampling. The selection of sampling locations and frequency in a monitoring plan plays a vital role in forming a valid data set for spatiotemporal model development. While the validation of our proposed model was limited by the available data set, it is important to highlight that our findings offer new insights into promoting synergy between sustainable monitoring and modeling, which is beneficial for building an early warning system for AMR issues in aquatic environments.

The RQ criteria employed were originally designed based on studies involving clear point source wastewater pollution loads into the environment. In the present study, where the pollution sources are putative and potentially nonpoint sources, such as hypothesized loads from sewer leaks, these assessed risk levels may not be directly applicable. The lack of precise and well-defined sources can introduce uncertainties and challenges in accurately assessing environmental risks. Therefore, any risk assessments and conclusions should be made with the recognition that the specific conditions of nonpoint source pollution may not align perfectly with the assumptions and criteria established for point source pollution, warranting further consideration and potentially different risk assessment approaches.

Our model, while centered on antibiotic and bacteria indicators as a pivotal factor in resistance development, does not overlook the complexities of antibiotic resistance mechanisms. It represents a deliberate focus, chosen to analyze a critical aspect of resistance in environmental contexts manageably. We acknowledge the multifaceted nature of resistance, involving genetic elements such as sul and tet genes and broader microbial interactions. Our approach, therefore, is not static but adaptive, and we are fully committed to evolving our model. Future research will integrate additional factors, enriching our understanding of the intricate dynamics of antibiotic resistance and enhancing the model’s applicability and depth. Nevertheless, the approach adopted in this study lays down a basic modeling framework to achieve the prediction of AMR impacts in a holistic manner. Furthermore, the model framework can be applied to other important antibiotics, bacterial pathogens, and ARB in the aquatic environment, including WHO priority ARB such as those resistant to the last resort antibiotics, that is, beta-lactam and carbapenem-resistant bacteria. In this study, the spatiotemporal distribution of the predicted antibiotics, bacteria, ARB, and environmental risks from antibiotics could be used as a benchmark to identify the hot spots of antibiotic resistance in the aquatic environment which in turn can help policymakers to develop an appropriate AMR risk management framework.^86^

Overall, the successful applications of our integrated modeling framework imply that with adequate support from the public (e.g., regulators, agencies, and nonprofit organizations) and private (e.g., researchers) sectors, more intensive sampling campaigns could be conducted to provide higher quality and sufficient data for the modeling work, thus enabling the development of a powerful toolbox to track environmental antibiotics, fecal bacteria, and potential AMR risks in the aquatic environments in the future.

## Supporting Information Available

Chemical detection and analysis; enumeration of bacteria; description of the hydrodynamic water quality model; PNEC value for environmental risk assessment; model calibration and validation; method recovery; key processes; key process parameters; PNEC values; statistical analysis; study area and modeling boundaries; calibrated results of the process-based model; modeling interactions (PDF)
