# Supplementary material for: Predicting Antibiotic Resistance and Assessing the Risk Burden from Antibiotics: A Holistic Modeling Framework in a Tropical Reservoir
Source: Environ Sci Technol. 2024 Apr 1;58(15):6781–92. doi: 10.1021/acs.est.3c10467 (PMC11025116; doi:10.1021/acs.est.3c10467)
Supplement: Supplementary file 1 — es3c10467_si_001.pdf [file es3c10467_si_001.pdf]

Supporting Information

Predicting antibiotic resistance and assessing the risk burdens from antibiotics: A holistic modelling framework in a tropical reservoir

Xuneng Tong[1], Shin Giek Goh[2], Sanjeeb Mohapatra[2], Ngoc Han Tran[2], Luhua You[2], Jingjie Zhang[2,3,4*], Yiliang He[5], Karina Yew-Hoong Gin[1,2*]

[1] Department of Civil & Environmental Engineering, National University of Singapore, 1 Engineering Drive 2, Singapore 117576, Singapore

[2] NUS Environmental Research Institute, National University of Singapore, 1 Create way, Create Tower, #15-02, Singapore 138602, Singapore

[3] Northeast Institute of Geography and Agroecology, Chinese Academy of Sciences, Changchun 130102, China

[4] Shenzhen Municipal Engineering Lab of Environmental IoT Technologies, Southern University of Science and Technology, Shenzhen, 518055, China

[5] School of Environmental Science and Engineering, Shanghai Jiao Tong University, Shanghai 200240, China

* Corresponding author, Tel: +65 6516 8104, email: ceeginyh@nus.edu.sg

*Co-corresponding author: lakejz@gmail.com

Contents of this file

Text of chemical analysis, bacteria enumeration, model description.

Table S1-S5.

Figure S1-S3.

1. Chemical detection and analysis

Water samples (1.0 L, i.e., 2 x 500 mL) were stored and transported in an ice box. 500 ml water samples were processed for each analysis, and 1 L of water samples was required for duplicate evaluations. After arriving in the lab, the water samples were shaken and then filtered through pre-weighed 0.7-µm glass fiber filters (Merck Millipore, Ireland) to remove suspended solids. Filtered water samples were adjusted to pH = 3 with HCl, and then spiked with 0.5 ppm isotopically labeled standard mix and 500 mg $Na_4EDTA.2H_2O$. Antibiotics in dissolved phase were detected and analyzed via solid-phase extraction (SPE) and ultrasound-assisted extraction, followed by high-performance liquid chromatography with tandem mass spectrometry (HPLC-MS/MS). Oasis HLB SPE cartridges (Waters, USA) were used to extract the analytes. The cartridges were conditioned with 5 mL methanol (HPLC level) and 5 ml acidified Milli-Q water (pH=3). Prepared water samples were loaded through the cartridges at a flow rate of 5 mL/min using a vacuum manifold. The cartridge was then washed with 5 ml methanol and 5 ml methanol-acetone (1:1, V/V) into a 15 mL tube at a flow rate of around 1 mL/min. Subsequentially, 10 mL samples were dried to almost dryness under a gentle nitrogen steam at 35 ^{o}C. The dried extracts were re-dissolved with 1 mL methanol-Milli-Q water (1:1, V/V). Quantification of the antibiotic residues was conducted by HPLC-MS/MS. The method recoveries for SMX and TMP were 100.4±4.2 and 99.7±7.2%, respectively. None of the target compounds were detected in the blank samples. Method Detection Limit (MDL) and Method Quantification Limit (MQL) were defined as the lowest observable concentration of analytes in the spiked samples, in which the signal-to-noise ratios of 3 and 10 were obtained, respectively.[1] Information on method recovery, MDL and MQL of targeted antibiotics are summarized in Table S1.

2. Enumeration of bacteria

The abundance of *E. coli* (in units of most probable number (MPN)/100 mL) was measured with *Colilert*™ (IDEXX Laboratories, Inc., Westbrook, Maine), according to manufacturer's instructions. The culture-based method was used to screen for *E. coli* against cotrimoxazole (EC_SXT, CFU/100mL) which is an antibiotic made up of two different antibiotics (SMX and TMP). Selective media with antibiotics (TMP: SMX) were used to enrich for the aforementioned bacteria taxa. Water samples (from 10 mL to 1 L) were filtered through sterile 0.45 μm pore size cellulose acetate filters (Sartorius Pte Ltd., Singapore). The filter was then placed on the agar surface and incubated overnight. Colonies (CFU/100 mL) grown were enumerated to determine the relative abundance of bacteria resistant to each antibiotic.

3. Description of the hydrodynamic-water quality model (HWQM)

3.1 Boundary conditions

The open boundaries in the study area were located around five major tributaries that feed into the reservoir. There were 30 open boundaries from the catchment area; the detailed locations of the study are shown in Figure S1. The hydrological data (inflow/outflow rate and water level) were assigned to the boundaries to build the hydrodynamic model. Monitored water quality data, including antibiotics and bacteria, in the tributaries were used to set the boundary conditions. The initial water quality condition was first set based on observations and then a restart file was used after the model was run for one-year spin-up time in which the concentrations of all substances on the final time step of the water quality simulation were stored. Except for the gate and pump operation to control the water level in the reservoir, it was assumed that there were no effluent discharge points in the study area since wastewater was not discharged into the surface water. The

simulation period was one year from 1 October 2015 to 1 October 2016 and the time step was set as 2 mins. The daily output results were used to compare with the field data.

3.2 Hydrodynamic model

The water current was determined by the numerical solution of the three-dimensional incompressible Reynolds-averaged Navier-Stokes equations invoking the assumptions of Boussinesq and hydrostatic pressure:

$$\frac{\partial u}{\partial x}+\frac{\partial v}{\partial y}+\frac{\partial w}{\partial z}=0 \tag{S1}$$

The u and v momentum equations are as follows:

$$\frac{\partial u}{\partial t}+u\frac{\partial u}{\partial x}+v\frac{\partial u}{\partial y}+w\frac{\partial u}{\partial z}=-g\frac{\partial \eta}{\partial x}+v_h\left(\frac{\partial^2 u}{\partial x^2}+\frac{\partial^2 u}{\partial y^2}\right)+\frac{\partial}{\partial z}\left(v_V\frac{\partial u}{\partial z}\right) \tag{S2}$$

$$\frac{\partial v}{\partial t}+u\frac{\partial v}{\partial x}+v\frac{\partial v}{\partial y}+w\frac{\partial v}{\partial z}=-g\frac{\partial \eta}{\partial y}+v_h\left(\frac{\partial^2 v}{\partial x^2}+\frac{\partial^2 v}{\partial y^2}\right)+\frac{\partial}{\partial z}\left(v_V\frac{\partial v}{\partial z}\right) \tag{S3}$$

The vertical momentum equation was reduced to the hydrostatic pressure assumption:

$$\frac{\partial p}{\partial z}=-\rho g \tag{S4}$$

where t is the time; x, y and z are the Cartesian co-ordinates; u, v, w are the velocity components in the x, y, and z direction; g is the gravitational acceleration; η is the water level; v_h and v_V are the kinematic eddy viscosity coefficients in the horizontal and vertical directions, respectively.

3.2. Advection-diffusion-reaction process

The model solved the advection-diffusion-reaction process on a predefined computational grid (cell). In this study, a mass-conservation method was used where the mass of modeled substance transported by flowing water from one cell to the next cell served as a negative term in

the first computational cell and as a positive term in the second computational cell. Therefore, the modeled substances could be transported through computational cells and hence, through the water system. All the necessary processes were accounted for in a computational cell, where substances could be transformed to other substances within a computational cell. The model calculates: (a) changes by transport including both advective and dispersive transport, respectively, where the flow of water and dispersion applied in the water quality model are derived from the hydrodynamic model; (b) changes by processes including physical processes such as reaeration and settling, (bio)chemical processes such as nitrification and denitrification as well as biological processes such as primary production and predation on phytoplankton by zooplankton; (c) changes by sources including waste loads and exchanges with boundaries. A representative equation of the advection-diffusion-reaction processes is shown:

$$M_i^{t+\Delta t} = M_i^t + \Delta t \times (\frac{\Delta M}{\Delta t})_{Tr} + \Delta t \times (\frac{\Delta M}{\Delta t})_P + \Delta t \times (\frac{\Delta M}{\Delta t})_S \quad \text{(S5)}$$

where Δt is the time step, M_i^t and $M_i^{t+\Delta t}$ is the simulated constituent mass at t and $t + \Delta t$, respectively. $(\frac{\Delta M}{\Delta t})_{Tr}$, $(\frac{\Delta M}{\Delta t})_P$ and $(\frac{\Delta M}{\Delta t})_S$ is the change by transport, physical-chemical-biological processes and sources, respectively.

3.3. Eutrophication module

The key processes in the eutrophication module include respiration, growth, mortality, settling and respiration of phytoplankton, cycling of nutrients such as mineralization, nitrification, denitrification and immobilization, and the production and use of dissolved oxygen. The eutrophication module includes the main driving factors and state variables as well as the necessary processes to describe the complex interactions between the different state variables, including nitrogen, phosphorus, silica and carbon cycling processes. Competition is based on a trade-off

principle between growth and requirement for the environment resources of nutrients and light through optimizing biomass under constraints of the concentration at the start of each time step, growth and mortality rates.[2] The key processes were summarized in Table S2. Representative calibrated values of the key kinetic processes are summarized in Table S3.

4. PNEC value for environmental risk assessment

The environmental risk assessment of antibiotic residues was considered in the light of two aspects: (1) development of AMR and (2) ecological toxicity to the aquatic ecosystem. For $PNEC_{AMR}$, data was obtained from the literature, with the definition of $PNEC_{Eco}$ as from previously.[3] Briefly, ecotoxicity data, including the lowest observed effect concentration (LOEC), no observed effect concentration (NOEC), half maximal lethal concentration (LC_{50}), half maximal inhibitory concentration (IC_{50}) or half-maximal effective concentration (EC_{50}) values combined with an assessment factor (AF), were used to calculate the $PNEC_{Eco}$:

$$PNEC_{Eco} = \frac{min(LOEC, NOEC, LC_{50}, IC_{50}, EC_{50})}{AF} \quad \text{(S6)}$$

where AF varies from 10 to 1000, depending on the nature of toxicological data. Generally, AF = 1000 is used for short-term ecotoxicological data (LC_{50}, IC_{50}, or EC_{50}) and AF = 100 is for long-term ecotoxicological data (LOEC or NOEC) with a relevant tested organism. The PNEC values for this study are shown in Table S4.

5. Model calibration and validation

To evaluate the model performance, the following statistical metrics were used: root-mean-square error (RMSE), the average standard deviation (ARD%) and Nash-sutcliffe coefficient (NSE).[4]

$$\mathrm{RMSE} = \sqrt{\frac{\sum (X_{Model} - X_{Field})^2}{N}} \quad \text{(S6)}$$

$$ARD\% = \frac{\sum |X_{Model} - X_{Field}| / X_{Field}}{N} \quad \text{(S7)}$$

$$\mathrm{NSE} = 1 - \frac{\sum (X_{model} - X_{field})^2}{\sum (X_{field} - \overline{X_{field}})^2} \quad \text{(S8)}$$

where X_{model} and X_{field} are the simulated and the observed values for the evaluated constituent, respectively; and the overbar denotes the temporal average. The NSE can range from $-\infty$ to 1. An efficiency of 1 corresponds to a perfect match of the model data to the observed data. An efficiency of 0 indicates that the model predictions are as accurate as the mean of the observed data, while an efficiency of less than 0 indicates that the observed mean is a better predictor than the model. The ARD calculates the absolute value of the relative difference between these two values, expressed as a percentage. This makes it a useful metric for understanding the error magnitude relative to the actual values. The thresholds in evaluating the model performance are not absolute and can be adjusted based on specific modeling objectives and the study context. The reason for defining these thresholds is to provide a standard way of interpreting the performance of models, allowing for comparison across different studies and applications. In this study, the thresholds for NSE (Nash-Sutcliffe Efficiency) and ARD (Absolute Relative Difference) were set in accordance with the standards established by Allen et al.[5] and Zhang et al.[6].

Table S1. Method recovery, MDL and MQL for antibiotic residues

Antibiotics	Recovery (%)	MDL (ng/L)	MQL (ng/L)
SXM	96±3	0.05	0.15
TMP	120±1	0.06	0.2

Table S2. Key processes in the HWQ model.

	Processes	Equations
Eutrophication Module	Phytoplankton growth	$Kgp_i = Kgp_i^0 \times Ktpg_i^T$
	Maintenance respiration	$Krsp_i = Krsp_i^0 \times Ktrsp_i^T$
	Mortality	$Kmrt_i = Kmrt_i^0 \times Ktmrt_i^T$
	Extinction	$k_d = k_b + k_{SPM} + k_{POM} + k_{Phy} + k_{HUM}$
	Nitrification	$nit = k_{nit} \times NH_4 \times f_{T,nit}$
	Denitrification	$den = k_{den} \times NO_3 \times f_{T,den}$
	Nitrogen adaption	$\sum_{i=1}^{n}(S_{N,i} * Phy_{i,\mathrm{new}}) \leq \sum_{i=1}^{n}(S_{N,i} * Phy_i) + NO_3 + NH_4$
	Phosphorus adaption	$\sum_{i=1}^{n}(S_{P,i} * Phy_{i,\mathrm{new}}) \leq \sum_{i=1}^{n}(S_{P,i} * Phy_i) + PO_4$
	Sedimentation	$sed_Y = \frac{v_Y - Y}{Z}$
	Yield	$\mathrm{Y} = Phy_i, POX$
	Grazer (1)	$grz_i = up \times Phy_i,$
	Grazer (2)	$grz_{POX} = up \times POX$
	Symbols in Eutrophication module	T is water temperature, Kgp_i is growth rate, Kgp_i^0 is growth rate at 0 °C, $Ktpg_i^T$ is temperature coefficient for growth; $Krsp_i$ is maintenance respiration rate, $Krsp_i^0$ is growth rate at 0 °C, $Ktrsp_i^T$ is temperature coefficient for maintenance respiration; $Kmrt_i^0$ is the specific mortality rate at 0 °C which is driven by salinity, kt_{mi} is temperature coefficient for mortality; k_d is total extinction coefficient; k_b is background extinction; k_{SPM} is extinction of inorganic suspended matter; k_{POM} is extinction of dead particulate organic matter; k_{Phy} is total extinction due to phytoplankton; k_{HUM} is extinction due to humic substances from freshwater input; *nit* is nitrification; *den* is denitrification; k_{nit} and k_{den} are nitrification and denitrification rate, respectively; $f_{T,nit}$ and $f_{T,den}$ are temperature function for nitrification and denitrification process, respectively; *Phy*i is phytoplankton type, $S_{X,i}$ is stoichiometry of nutrient; *sed* is settling; *v* is settling velocity; *X* is element carbon, nitrogen, phosphorus and silicate; *POX* is particulate organic element carbon, nitrogen,

		phosphorus and silicate; *grz* is grazing by filter feeders; *up* is uptake rate of organic matter by grazers.
Antibiotics Module	Overall degradation	$Rdeg = K0deg + (K1deg_i^T * Ktdeg_i^{(T-20)} * frdeg * Cant)1$
	Partition (1)	$LogKppoc = LogKppoc_i^{20} + a * (\frac{1}{T+273.15} - \frac{1}{293.15})$
	Partition (2)	$LogKpalg = LogKpalg_i^{20} + a * (\frac{1}{T+273.15} - \frac{1}{293.15})$
	Partition (3)	$K'_{ppoc} = 10^{logK_{ppoc}} * 10^{-6}$
	Partition (4)	${K_{palg}}' = 10^{logK_{palg}}$
	Fraction of partition (1)	$f_d = \frac{\phi}{\phi + K'_{ppoc} * (C_{poc} + X_{doc} * C_{doc}) + K'_{palg} * C_{alg}}$
	Fraction of partition (2)	$f_{doc} = (1 - fd) * \frac{K'_{ppoc} * X_{doc} * C_{doc}}{K'_{ppoc} * (C_{poc} + X_{doc} * C_{doc}) + K'_{palg} * C_{alg}}$
	Fraction of partition (4)	$f_{poc} = (1 - fd) * \frac{K'_{ppoc} * C_{poc}}{K'_{ppoc} * (C_{poc} + X_{doc} * C_{doc}) + K'_{palg} * C_{alg}}$
	Fraction of partition (5)	$f_{alg} = (1 - f_d - f_{doc} - f_{poc})$
	Symbols in antibiotics module	$K0deg$ is zeroth order degradation rate, $K1deg_i^T$ is the first order degradation rate,$Ktdeg_i^{(T-20)}$is temperature coefficient of degradation, T is temperature, $frdeg$ is fraction subjected to degradation, $Cant$ is total antibiotics concentration, ϕ is porosity; ${K_{palg}}'$ and ${K_{ppoc}}'$ are the partition coefficient for algae and dead particulate organic matter, respectively; C_{alg} C_{poc}, C_{doc} are the concentration of algae biomass, POC, DOC respectively.
Bacteria module	Overall decay	$CF_{decay} = K_M * C_{CF}$
	Overall Mortality	$K_M = (K_B + K_{Cl}) * K_T^{(T-20)} + K_R$
	Chloride-dependent mortality	$K_{Cl} = k_{Cl} * C_{cl}$
	Solar radiation-dependent mortality	$K_R = k_{rd} * DL * f_{uv} * I_0 \frac{(1 - e^{-\varepsilon H})}{\varepsilon H}$

	$$\varepsilon = \frac{1.8}{SD}$$
Symbols in bacteria module	CF_{decay} is the concentration of bacteria over time, K_M is the mortality rate (generalized as the first-order), C_{CF} is the bacteria concentration; K_B is the basic mortality rate; K_{Cl}, K_T and K_R is the dependent mortality rate of chloride, temperature and solar radiation, respectively; k_{Cl} and C_{cl} is the chloride-dependent mortality constant and chloride concentration, respectively; k_{rd} is the solar radiation-dependent mortality rate, DL is the day-length, f_{uv} is the fraction of light, I_0 is the solar radiation at the water surface, ε is the extinction of light, H is water depth; SD is the Secchi disk depth.

Table S3. Key process parameters of the water quality model.

	Parameters	Description	Calibrated Value	Unit	Source
Eutrophication Module	FrAutFDI	Autolysis fraction of mortality	0.35	-	[7]
	FrDetFDI	Detritus fraction by mortality	0.55-0.62	-	[7]
	DMCFFDI	DM:C_ratio	2.5	-	[7]
	NCRFDI	Stocheometry_of_nutrient_N	0.125-0.275	-	[7]
	PCRFDI	Stocheometry_of_nutrient_P	0.01-0.03	-	[7]
	SCRFDI	Stocheometry_of_nutrient_Si	0.0018-0.66	-	[7]
	ChlaCFDI	Stocheometry_of_chlorphyll	0.02-0.04	-	[7]
	PPMaxFDI	Maximum_primary_production	0.16-0.431	-	[7]
	TcPMxFDI	Temp._coeff._growth_processes	1.059-1.0985	-	[7]
	MRespFDI	Maintenance_respiration_rate	0.012-0.031	-	[7]
	TcRspFDI	Temperature_coeff._respiration	1.072	-	[7]
	Mort0FDI	salinity_dependent_mortality_rate	0.19-0.54	-	[7]
	TcMrtFDI	Temperature_coeff._for_mortality	1.08	-	[7]
	VSedFDI	Sedimentation rates phytoplankton	0.75	m/day	[7]
Antibiotics Module	lKpocSMX	Log Partition Coefficient SMX-dead particulate organic matter (POC) in the water column	3.50	Kg^{-1} C	EPI Suite^{TM}, Measured

	lKphySMX	Log Partition Coefficient SMX-Phytoplankton (ALG) in the water column	3.50	Kg^{-1} C	EPI Suite™, Measured
	lKpocSMXS1	Log partition coefficient for SMX-POC in the sediment layer	2.60	Kg^{-1} C	EPI Suite™, Measured
	lKphySMXS1	Log partition coefficient for SMX-ALG in the sediment layer	2.60	Kg^{-1} C	EPI Suite™, Measured
	XDOCBPA	Efficiency dissolved organic matter (DOC) relative to POC for SMX	0.18	-	[8]
	RcSMX	Degradation rate of BPA	0.013	d^{-1}	EPI Suite™
	lKpocTMP	Log Partition Coefficient TMP-POC in the water column	3.55	Kg^{-1} C	EPI Suite™, Measured
	lKphyTMP	Log Partition Coefficient TMP-ALG in the water column	3.55	Kg^{-1} C	EPI Suite™, Measured
	lKpocTMPS1	Log partition coefficient for TMP-POC in the sediment layer	3.45	Kg^{-1} C	EPI Suite™, Measured
	lKphyTMPS1	Log partition coefficient for TMP-ALG in the sediment layer	3.45	Kg^{-1} C	EPI Suite™, Measured
	XDOCTMP	Efficiency dissolved organic matter (DOC) relative to POC for TMP	0.18	-	[8]
	RcTMP	Degradation rate of TMP	0.013	d^{-1}	EPI Suite™
	SWPORH	Porosity in the top sediment layer	0.66	-	Measured
Bacteria Module	RcMrtEColi	Basic mortality rate	0.8	d^{-1}	[8]
	TcMrtEColi	Temperature coefficient of the mortality rate	1.07	-	[8]
	SpMrtEColi	Chloride coefficient of the mortality rate	0.001	m^{3}g^{-1}d^{-1}	[8]
	CFRAD	Radiation dependent mortality constant	0.086	m^{3}W^{-1}d^{-1}	[8]

Table S4. PNEC values used in this study**.**

Risk type	Antibiotics	Species	Endpoint	Critical effects	PNEC	Source
Ecological	SMX	*P.subcapitata*	NOEC	Growth inhibition	59	9
	TMP	*A. cylindrica*	EC_{50}	Growth inhibition	32	10
AMR	SMX	-	-	-	16000	11
	TMP	-	-	-	500	11

Table 5. Statistic analysis of multi-linear regression.

	t value	p-value	lower 95%	upper 95%
Intercept	-0.696	0.502	-0.879	0.460
SMX	-2.021	0.071	-0.248	0.012
TMP	1.861	0.092	-0.082	0.912
$\log_{10}$ (*E. coli*)	8.063	0.000	0.447	0.788

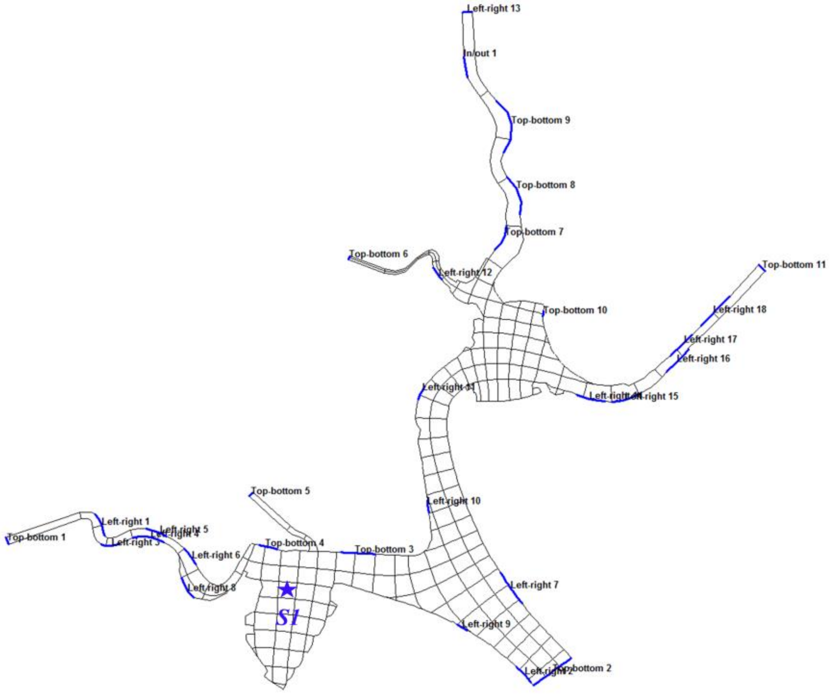

Figure S1. Study area and the modelling boundaries.

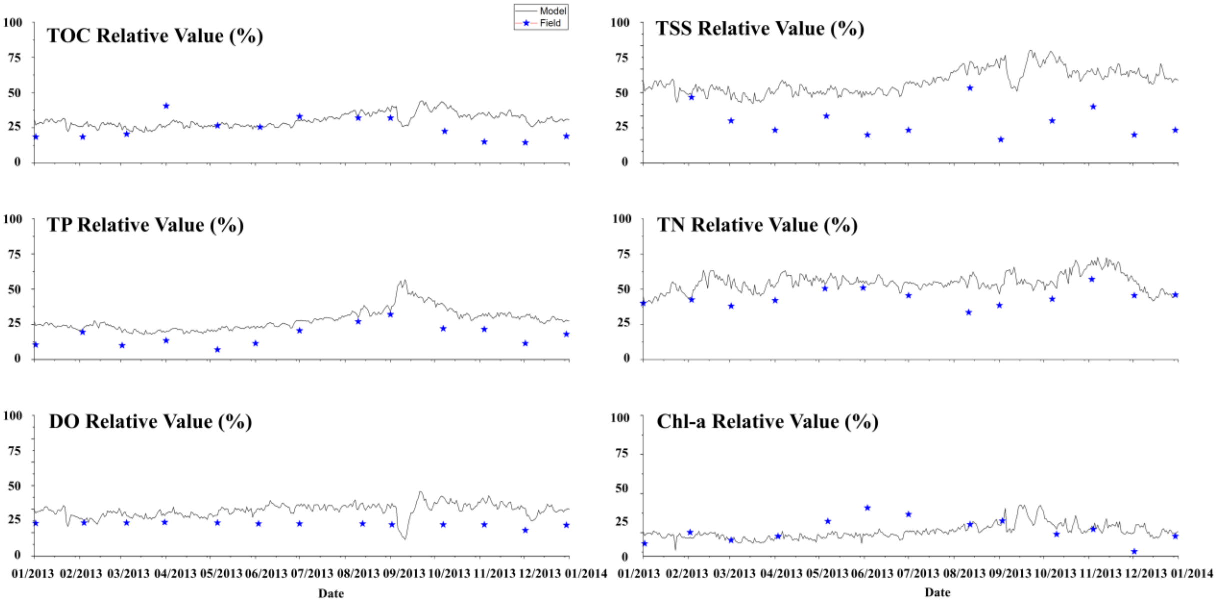

Fig. S2. Calibrated results of process-based model; The data can be found in previous study.[12, 13] Relative Value: Observed results relative to the maximum threshold value set by the local authority.

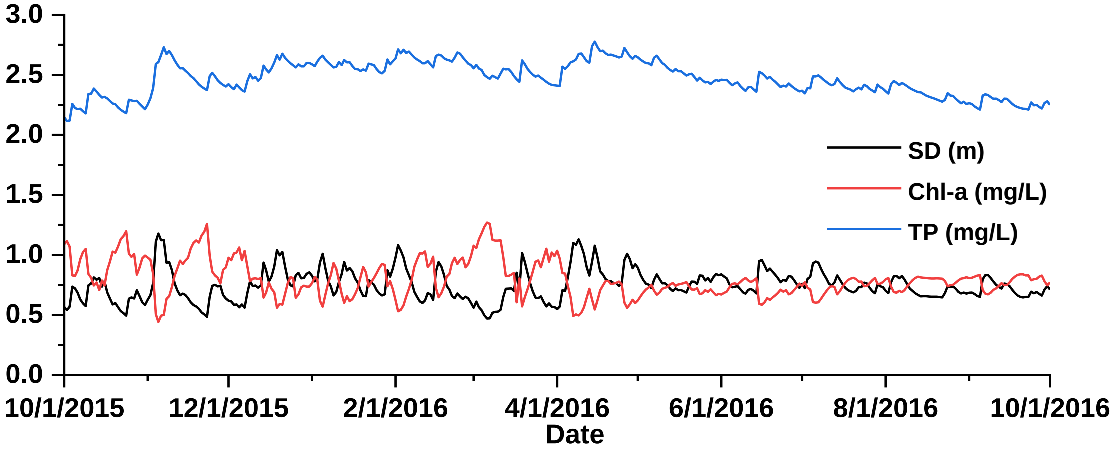

Figure S3. Modeling interactions between Secchi disk depth, Chlorophyll-a and Total phosphorus.

References

1. Tran, N. H.; Chen, H.; Van Do, T.; Reinhard, M.; Ngo, H. H.; He, Y.; Gin, K. Y.-H., Simultaneous analysis of multiple classes of antimicrobials in environmental water samples using SPE coupled with UHPLC-ESI-MS/MS and isotope dilution. *Talanta* **2016,** *159*, 163-173.

2. Los, F. J., *Eco-hydrodynamic modelling of primary production in coastal waters and lakes using BLOOM*. Ios Press: 2009; Vol. 1.

3. Tran, N. H.; Hoang, L.; Nghiem, L. D.; Nguyen, N. M. H.; Ngo, H. H.; Guo, W.; Trinh, Q. T.; Mai, N. H.; Chen, H.; Nguyen, D. D., Occurrence and risk assessment of multiple classes of antibiotics in urban canals and lakes in Hanoi, Vietnam. *Science of The Total Environment* **2019,** *692*, 157-174.

4. Nash, J. E.; Sutcliffe, J. V., River flow forecasting through conceptual models part I—A discussion of principles. *Journal of hydrology* **1970,** *10* (3), 282-290.

5. Zhang, W.; Watson, S. B.; Rao, Y. R.; Kling, H. J., A linked hydrodynamic, water quality and algal biomass model for a large, multi-basin lake: a working management tool. *Ecological modelling* **2013,** *269*, 37-50.

6. Allen, J.; Somerfield, P.; Gilbert, F., Quantifying uncertainty in high-resolution coupled hydrodynamic-ecosystem models. *Journal of Marine Systems* **2007,** *64* (1-4), 3-14.

7. Hydraulics, D., Delft3D-WAQ Users Manual. WL, Delft Hydraulics, Delft, The Netherlands: 2009.

8. Karickhoff, S. W.; Brown, D. S.; Scott, T. A., Sorption of hydrophobic pollutants on natural sediments. *Water research* **1979,** *13* (3), 241-248.

9. Yang, L. H.; Ying, G. G.; Su, H. C.; Stauber, J. L.; Adams, M. S.; Binet, M. T., Growth-inhibiting effects of 12 antibacterial agents and their mixtures on the freshwater microalga

pseudokirchneriella subcapitata. *Environmental Toxicology and Chemistry: An International Journal* **2008,** *27* (5), 1201-1208.

10. Ando, T.; Nagase, H.; Eguchi, K.; Hirooka, T.; Nakamura, T.; Miyamoto, K.; Hirata, K., A novel method using cyanobacteria for ecotoxicity test of veterinary antimicrobial agents. *Environmental Toxicology and Chemistry: An International Journal* **2007,** *26* (4), 601-606.

11. Bengtsson-Palme, J.; Larsson, D. J., Concentrations of antibiotics predicted to select for resistant bacteria: Proposed limits for environmental regulation. *Environment International* **2016,** *86*, 140-149.

12. Tong, X.; You, L.; Zhang, J.; Chen, H.; Nguyen, V. T.; He, Y.; Gin, K. Y.-H., A comprehensive modelling approach to understanding the fate, transport and potential risks of emerging contaminants in a tropical reservoir. *Water Research* **2021,** *200*, 117298.

13. Tong, X.; You, L.; Zhang, J.; He, Y.; Gin, K. Y.-H., Advancing prediction of emerging contaminants in a tropical reservoir with general water quality indicators based on a hybrid process and data-driven approach. *Journal of Hazardous Materials* **2022,** *430*, 128492.
